# The Investment in Scent: Time-Resolved Metabolic Processes in Developing Volatile-Producing *Nigella sativa* L. Seeds

**DOI:** 10.1371/journal.pone.0073061

**Published:** 2013-09-03

**Authors:** Wentao Xue, Albert Batushansky, David Toubiana, Ilan Botnick, Jedrzej Szymanski, Inna Khozin-Goldberg, Zoran Nikoloski, Efraim Lewinsohn, Aaron Fait

**Affiliations:** 1 The Jacob Blaustein Institutes for Desert Research, Ben-Gurion University of the Negev, Midreshet Ben-Gurion, Israel; 2 Newe Ya’ar Research Center, Agricultural Research Organization, Ramat Yishay, Israel; 3 Max-Planck Institute of Molecular Plant Physiology, Potsdam, Germany; RIKEN PSC, Japan

## Abstract

The interplay of processes in central and specialized metabolisms during seed development of *Nigella sativa* L. was studied by using a high-throughput metabolomics technology and network-based analysis. Two major metabolic shifts were identified during seed development: the first was characterized by the accumulation of storage lipids (estimated as total fatty acids) and N-compounds, and the second by the biosynthesis of volatile organic compounds (VOCs) and a 30% average decrease in total fatty acids. Network-based analysis identified coordinated metabolic processes during development and demonstrated the presence of five network communities. Enrichment analysis indicated that different compound classes, such as sugars, amino acids, and fatty acids, are largely separated and over-represented in certain communities. One community displayed several terpenoids and the central metabolites, shikimate derived amino acids, raffinose, xylitol and glycerol–3-phosphate. The latter are related to precursors of the mevalonate-independent pathway for VOC production in the plastid; also plastidial fatty acid 18∶3n-3 abundant in “green” seeds grouped with several major terpenes. The findings highlight the interplay between the components of central metabolism and the VOCs. The developmental regulation of *Nigella* seed metabolism during seed maturation suggests a substantial re-allocation of carbon from the breakdown of fatty acids and from N-compounds, probably towards the biosynthesis of VOCs.

## Introduction

During seed development, carbon metabolism is committed to three different directions, namely, accumulation of storage reserves, preparation for germination, and acquisition of desiccation tolerance [1]–[3]. In parallel to driving the development of seeds on the mother plant, central carbon metabolism provides the building blocks for the production of specialized metabolites, including: fatty acids, pigments, phenolic compounds, and alkaloids, as well as volatile organic compounds (VOCs) in VOC-producing seeds. The seeds of most species do not commonly accumulate volatiles, but those of the Brassicaceae and of some other families do accumulate non-volatile glucosinolates, the precursors of sulfur volatiles, which are degraded into volatile compounds upon tissue disruption [4]. The so-called “seeds” that accumulate essential oils in species like fennel, caraway and anise are in fact mericarps (fruits) [5]. In contrast, *Nigella sativa* L. (Ranunculaceae), popularly known as black cumin, accumulates essential oil in its true seeds, thus providing a model system to study the inter-regulation between the production of VOCs and the accumulation of the storage reserves that are characteristic of seed development and maturation [6].

*Nigella* seeds have been widely used since antiquity both as a medicine and as a spice in the Middle East, India and Europe [7]. These seeds contain major pharmacoactive components, including the monoterpene thymoquinone, the saponin α-hederin, and unique alkaloids [7]. The seeds also contain relatively high levels of fixed oil, triacylglycerols composed mainly of unsaturated fatty acids (oleic and linoleic acids), palmitic acid and, unusually, eicosadienoic acid (20∶2n-6), which rarely accumulates in seeds [7]–[10]. Although the genetics underlying the production of the VOCs has been documented, knowledge of the biochemistry of the volatiles, which include more than 30,000 compounds, remains fragmented [11], [12]. Moreover, profiles of volatiles can change swiftly as a consequence of environmental and herbivorous pressure or in response to developmental cues [13], [14]. Therefore, it is likely that there is a highly dynamic interplay between central metabolism and the biosynthesis of volatiles. We hypothesize that during the period of seed development, which generally requires tight regulation of metabolic processes [15]–[17], there is probably a balance between incorporation of carbon and nitrogen into storage reserves and production of volatiles.

The recent development of advanced analytic tools enables comprehensive phenotyping of plant tissue and molecular characterization of developmental processes [1]. The metabolic phenotyping of seeds has aided in the description and identification of the processes central to seed physiology [17]–[19]. To understand the organization of relational ties between metabolites, reflecting not only substrate-product relationships but also regulatory effects, one may apply various similarity measures to (normalized) metabolic profiles. The resulting similarity matrices can, in turn, be effectively used to generate hypotheses and descriptive analyses of metabolism [20], [21]. The analysis of the relationships between time-resolved profiles is usually performed by applying symmetric similarity measures (e.g., Pearson, Spearman, and partial correlation), eventually extracting *undirected* relationships [22]. However, cellular networks spanning different molecular levels (e.g., gene regulation, signaling, and metabolism) are in fact inherently *directed,* implying the existence of driving and responding biochemical entities (e.g., feedback regulation, transcription factors and signaling proteins). The use of similarity measures for determining directed (causal) relationships is precluded by the need for very long time-series data, exceeding 100 time points [23].

Here, we gathered and analyzed, using different analytical platforms, metabolite data sets from developing VOC-producing seeds of *Nigella*. We then integrated the time series metabolite dataset via a network-based analysis to study the coordinated interplay between the metabolisms of volatile and non-volatile products during seed development. To this end, we employed a recently introduced similarity measure to identify directed coordinated patterns of change between metabolites during seed development [24]. The results are discussed against the background of the current understanding of seed metabolism and of the biosynthesis of volatiles.

## Materials and Methods

### Chemicals

All chemicals were purchased from Sigma-Aldrich Israel Ltd. (Jerusalem, Israel) with the exception of N-methyl-N-[trimethylsilyl] trifluoroacetamide (Macherey-Nagel GmbH & Co. KG, Düren, Germany).

### Plant Growth and Seed Collection

*Nigella sativa* L. seeds, accession Ein Harod (*EH*) from Ein Harod in Israel (latitude: 32° 33′ 00′′ N; longitude: 35° 23′ 00′′ E [7]) were collected from plants grown in open field conditions at Newe Yaar Research Center in Northern Israel. Plants were drip irrigated and fertigated using commercially accepted practices. Twenty milligrams of seeds were collected from three individual plants (n = 3) at 15 time points during their development from anthesis [0 days after anthesis (DAA)] to mature seeds (75–85 DAA).

### Extraction and Analysis of Total Protein and Chlorophyll-a Content

Total protein was determined by the Bradford method [25] using Protein Assay reagent (Sigma-Aldrich Israel Ltd., Jerusalem, Israel). Protein extraction was performed by crushing the seeds with 0.1 M NaOH at 95°C for 1 h. The samples were mixed with the reagent and measured at 595 nm after incubation at room temperature. To measure the amount of chlorophyll-*a* (Chl-a) seeds were crushed, placed in 80% ethanol, and then held at 4°C for two days in tightly closed tubes in the dark. The chlorophyll-*a* content was estimated from the measurement of the supernatant absorbance at 665 nm by using the equation Chl-a, in ug/mg = (OD_665_*13.9)*2/weight [26].

### Extraction, Derivatization and Analysis of Primary Metabolites Using GC-MS

Material collected as described above was extracted according to the protocol described in Lisec et al. [27] and analyzed using a sqGC-MS (Thermo Scientific Ltd) by adjusting the extraction protocol to seed material, as described in Fait et al. [17]. Relative metabolite content was calculated as described in Roessner et al. [28] following peak identification using Xcalibur software. Metabolites were annotated by comparison to mass spectra in the NIST library and the Golm database [29], [30].

### Extraction and Analysis of Fatty Acids

Seeds were ground and transmethylated with 2% H_2_SO_4_ in dry methanol (v/v) at 70°C for 1 h. Heptadecanoic acid (C17∶0) was added as the internal standard. Gas chromatographic analysis was performed according to Cohen et al. [31]. Fatty acid methyl esters were identified by co-chromatography with authentic standards.

### Multivariate and Statistical Analysis

Principal component analysis (PCA) was performed on the data sets obtained from metabolite profiling with the software package tMEV [32]. Prior to the analysis, data were log-transformed and normalized to the median of the entire sample set for each metabolite. Differences between means were tested for significance by the sum of squares simultaneous test procedure (SS-STP) [33] to reduce the number of multiple tests. Hypothesis testing was carried out at significance level of 0.05. Model-based clustering was conducted by using the “cluster” package in R version 2.15.1. The Bayes information criterion was used to determine the number of clusters.

### Directed Network Generation

For directed network generation, we used the recently introduced asymmetric measure, known as *iota* (denoted by *i*). Iota is a permutation-based measure, which relies on sorting a time series in increasing order and quantifying how the implied order affects the monotonicity of the remaining time series [24], [34]. The monotonicity in a re-ordered time series, based on the order-inducing permutation of the other, is quantified by the normalized number of crossing points. For instance, two time series, illustrated in the upper left corner Figure S2, are denoted as red and black. Sorting of the black time series in increasing order induces a re-ordering of the red time series, which results in 4 crossing points and a value for iota of 4/10 (where 10 is the maximum number of crossings in a time series on 6 time points). If this value is statistically significant, then a directed edge is established originating in the node described by the black time series and ending in the node whose behavior is characterized by the red time series. To reconstruct the network, we first determined the threshold value for *i* to ensure a *q*-value of 0.05. For a threshold value τ*_i_*, the *q*-value is defined as the minimum false discovery rate (FDR) attained at or above the given threshold score. The *q*-value can be readily determined from an empirically estimated null-distribution. Here, the null distribution is obtained by 500 shuffling of the profiles independently of each other, followed by re-estimation of the iota values. For the case of the *Nigella* metabolomics data set, there are 100 metabolites, for which the threshold value τ*_i_* = 0.966 implies a *q*-value of 0.05. Finally, the network in which the nodes represent metabolites includes directed edges only for those pairs whose *i* value is above the threshold τ*_i_*. The directed edges can be regarded as capturing putative substrate-product and regulatory relationships as well as the dependence between biochemical pathways.

To further reveal the clustering structure of this network, communities were determined by performing short random walks on the network and imposing a limit of *k* = 20 on the number of nodes in each of the identified communities. The idea behind this procedure is that short random walks tend to remain in the same community. The robustness of the community results was established by varying the parameter *k* in the interval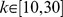. The resulting communities were visualized using Cytoscape version 2.8.3.

## Results

**Figure 1:**
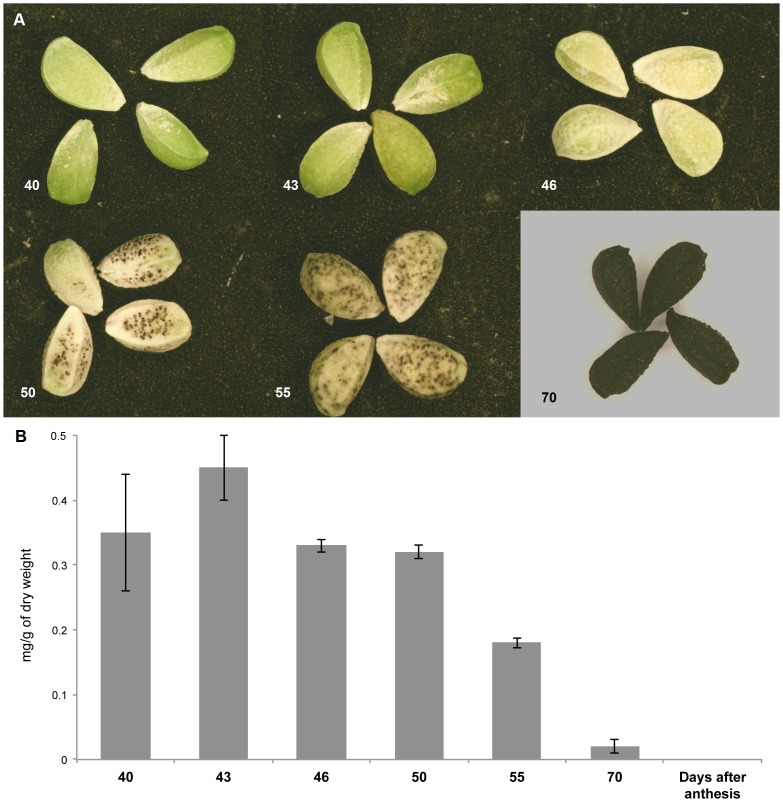
Phenotype (A) and chlorophyll content (B) of *Nigella sativa* developing seed from 40^th^ to 70^th^ DAA.

*Nigella* seeds remain green up to 46 DAA; at 55 DAA the color turns gradually to black (Figure 1A). The content of chlorophyll *a* at late maturation (Figure 1B) suggests that light reactions are taking place, probably reaching a maximum at 43 DAA and subsequently decreasing gradually to the minimum observed at 70 DAA. After this time, seeds lose chlorophyll and acquire black pigmentation (Figure 1B).

### Metabolic Profiling Analysis of *Nigella* Seeds Identifies Distinct Developmental Milestones in Central and Specialized Metabolism

To investigate the regulation between central metabolites during seed development, we utilized an established gas chromatography-mass spectrometry (GC-MS)-based protocol [28]. The relative contents of over 70 annotated metabolites from seeds at 14 different time points from early, through mid to late development were quantified (Materials and Methods). The resulting data set is presented in Table S1.

**Figure 2:**
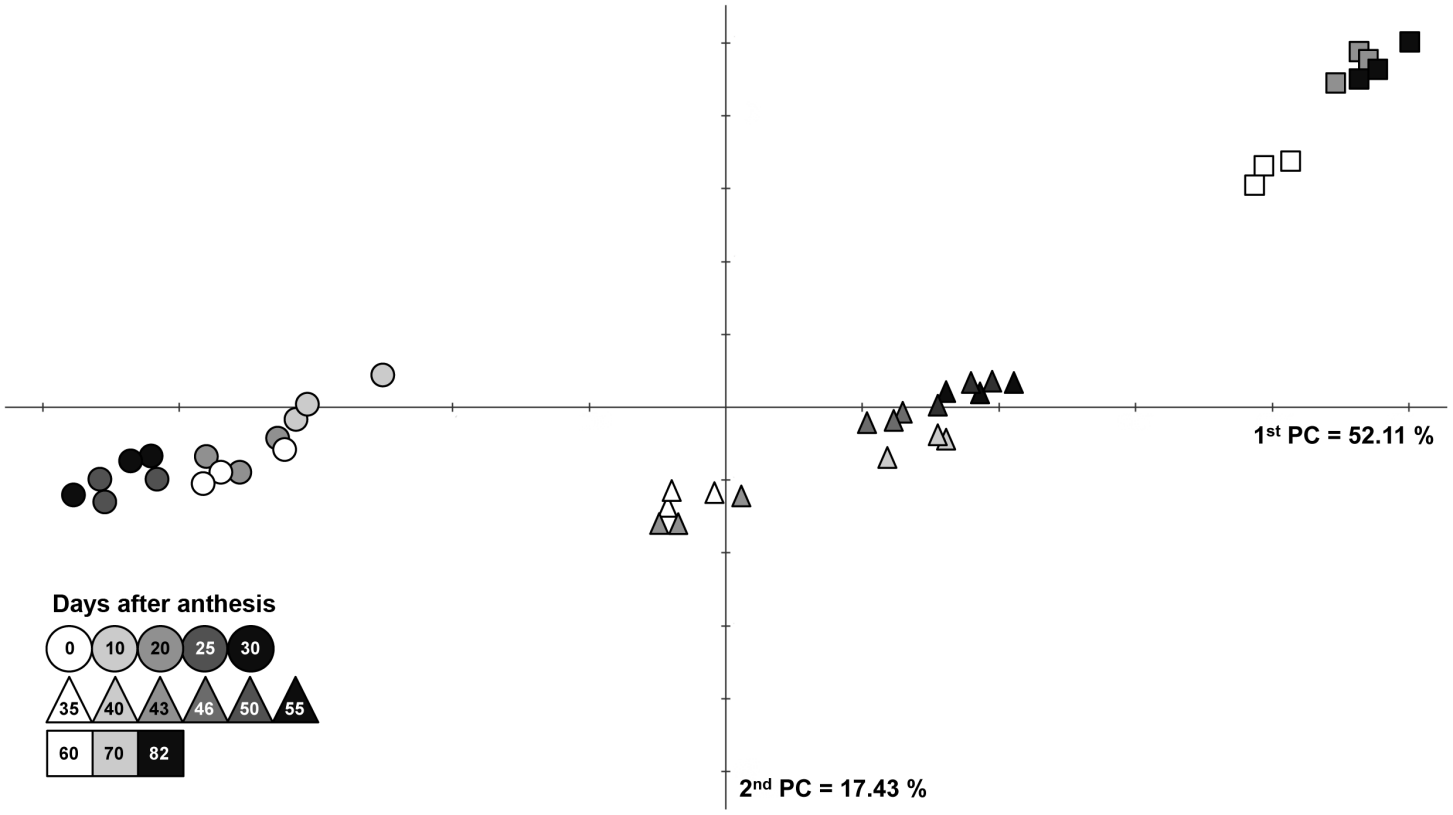
Principal component analysis of central metabolites data during seed development in DAA (see legend). Plot represents 1^st^ (X-axis) and 2^nd^ (Y-axis) principal components. Variance explained by each component is indicated in brackets. Shapes represent different developmental clusters.

**Figure 3:**
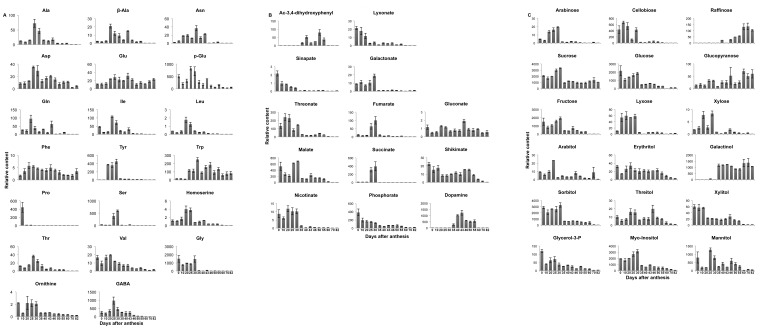
Relative content of central metabolites identified by GC-MS analysis of *Nigella* seeds during development. A – relative content of amino acids, B – relative content of carboxylic and other acids, C – relative content of sugars, sugar alcohols and others. Each bar represents the mean values of three replicates±SE.

The Bayes information criterion in combination with model-based clustering of the developmental time series (Materials and Methods) was used to estimate the number of clusters, with each cluster exhibiting similar metabolite profiles distinct from the other samples occupying different clusters. This resulted in three clusters (Figure S1), in line with the dispersion suggested by the PCA (Figure 2, Table S3), which shows that samples from early, mid, and late maturation belong to different clusters. The three clusters corresponding to the different developmental periods are characterized by two distinct shifts in metabolite abundance. By using the conservative SS-STP [33] to test for differences between groups of samples, we identified the metabolites that contribute significantly to the shift between the determined clusters (Table S3). The first shift occurs at 35 DAA, and it is characterized by a significant drop in the content of sugars and glycolysis intermediates, of the triacylglycerol precursor glycerol–3-phosphate, and of myo-inositol, malate and nicotinate. Exceptions among the sugars were galactinol, raffinose and sucrose, whose abundance increased significantly. Among the N-compounds, Asn (at 35 DAA) followed by dopamine (at 35–40 DAA), displayed an exceptionally high, but transient, accumulation during this period [10 and 1000-fold change, respectively, Figure 3A–C)], although the majority of amino acids had increased transiently earlier in development (25 DAA). The second shift from 55 to 60 DAA was characterized by decreased contents not only of the hexoses and sugar alcohols, but also of shikimate and the shikimate-pathway-related metabolites 3,4-dihydroxyphenyl-acetate, dopamine and beta-Ala. In addition, an increase in raffinose, a dehydration-associated sugar, was prominent at this stage (Table S3).

Metabolites contributing to the overall distribution of the samples on the principal components (PCs) were derived from the loadings of the first three components and include the sugars galactinol, raffinose, glucopyranose and cellobiose, together with 3,4-dihydroxyphenyl-acetate, glycerate, threonate 1,4-lactone, succinate, nicotinate, malate, fumarate, and the amino acids Ser, Pro, Gln, GABA, and Tyr and the related catecholamine, dopamine (Table S2). Analysis of the pattern of changes of the individual metabolites across the different developmental stages resulted in the trends depicted in Figure 3A–C, as described in the following sections.

#### Glycolysis and sugar metabolism

Most glycolytic intermediates and other sugars displayed an increase between day 10 and day 25–30, followed by an abrupt decrease. The above patterns of change was characterized by a sharp increase in abundance of these sugars as well as in arabinose, lyxose and xylose between 20 and 30 DAA. The general decrease in sugars was coupled to the accumulation of galactinol and raffinose, which are desiccation-associated sugars. Raffinose increased 100-fold, a finding that emphasizes the functional relevance of this sugar in seed late maturation as suggested for *Arabidopsis* [16], [17], [35].

#### TCA cycle and amino acid metabolism

TCA cycle intermediates such as succinate, fumarate and, to some extent, malate, as well as the associated metabolite GABA where shown to increase markedly between 25 and 35 DAA. In contrast, the levels of itaconate, a precursor of aconitate, dropped dramatically following anthesis and then increased transiently at 25 DAA and between 35 and 43 DAA. The increased activity of the TCA cycle, reflected by the abundance of TCA intermediates, was associated with the production of pyruvate-derived 2-isopropyl malate, a precursor of the amino acids Val, Leu and Ile. Similarly to itaconate, 2-isopropyl malate was shown to transiently but dramatically increase at 25 DAA and later at 43 DAA, a time point that was also characterized by major changes in N-compounds (see below).

#### Amino acids

Rapid changes characterized the level of amino acids in the *Nigella* seeds during development. The contents of the amino acids Val, Leu, Ile, Thr and particularly of Tyr and Ser, increased transiently by more than 10-fold between 20 and 30 DAA. The majority of the amino acids, including Asp, Glu, Ala, β-Ala, Ser and homo-Ser, accumulated at 25 DAA. Stages subsequent to 30 DAA were characterized by a gradual but steady decrease in the content of all amino acids, with the exception of Asp, Asn, Ala, Val, and β-Ala and to a lesser extent Glu and Gln. The latter showed a second transient increase in content around 43–46 DAA (Figure 3A).

A succession of changes in the content of different amino acids during seed development could be indicative of inter-convertibility between amino acids. For example, Pro showed the most pronounced change of a 20-fold decrease in abundance between the first and second sampling dates, probably as a result of its catabolism to Glu. The content of Pro continued to decrease significantly for the next 2 time points. Increases in Asn and particularly in Gln preceded the first wave of general increases in amino acid content; at day 20, the abundances of Gln and Asn increased transiently (by 5-fold and 2-fold, respectively) and then decreased to initial levels at the subsequent time points (25 and 30 DAA). For the N-containing compounds, the content of Asp-derived nicotinate doubled at 20 DAA and dropped by fivefold at 35 DAA, when comparatively sharp increases in dopamine, Asn, Ala-CO_2_ and Trp were measured (Figure 3A). Moreover, during this period, the nicotinate-derived compound 6 hydroxy nicotinate accumulated by about 25-fold in comparison to its level during early development. At the very end of the developmental period under investigation, namely, between 70 to 82 DAA, significant accumulation of the amino acids Glu, Asp, and Ala-CO_2_ was found in the dry seeds.

### Shikimate-associated Changes

An increase in the abundance of Trp followed that of Phe and Tyr at 20–30 DAA. As seed development proceeded, Trp showed additional peaks in relative content at 46 DAA. Major changes were also observed for shikimate-derived dopamine and for Phe-derived 3,4-dihydroxyphenyl-acetate from 40–43 DAA to 55 DAA. Shikimate, a precursor of chorismate, accumulated transiently during early seed development at 0–25 DAA and later between 46 and 55 DAA. Chorismate-derived 4-amino-benzoate (anthranilate) was decreased gradually throughout seed development. The cinnamate derived 4-hydroxy-benzoate (from Phe metabolism) displayed a 10-fold increase at the end of seed maturation (70 DAA).

### Fatty Acids Analysis of *Nigella* Seeds

**Figure 4:**
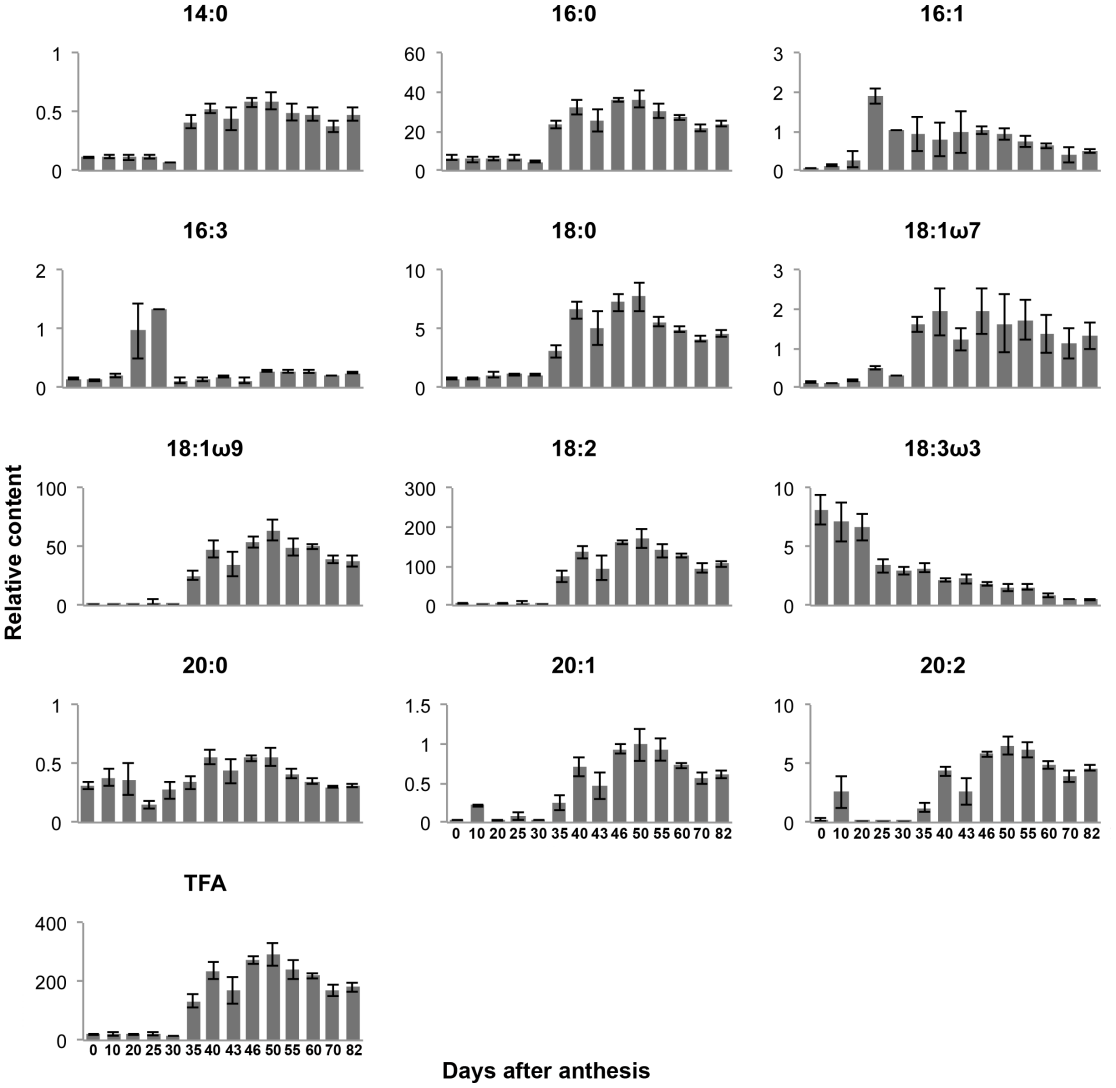
Fatty acid content of *Nigella* seeds during development. The unit of the Y-axis is mg g^–1^ fresh weight. Each bar represents the mean values of three replicates ±SE.

The composition of fatty acids was determined in mature *Nigella* seeds from the representative accession (*EH*), and the relative proportions [expressed as percentage of total fatty acids (TFA)] and absolute concentrations of fatty acids (µg per gram of seeds) were determined in *Nigella* seeds at different stages of their development (Figure 4, Table 1). Both the polyunsaturated fatty acid α-linolenic acid (18∶3n-3), a substrate of lipoxygenase [LOX (linoleate:oxygen oxidoreductase, EC 1.13.11.12)], and C_16_ unsaturated fatty acids were present during early stages of seed maturation (“green” stage) when the immature seeds were still rich in photosynthetic pigments (Figure 1A). These unsaturated fatty acids are components of the chloroplast membrane lipids of flowers and of “green developing” seeds. Of note was the sharp decrease in the concentration of the 18∶3n-3 fatty acid between 20 and 25 DAA and its continued low levels throughout maturation. In contrast, the rapid increase in TFA content, which had occurred by 35 DAA, was accompanied by the accumulation of the polyunsaturated fatty acids 18∶1n–9 and 18∶2n–6, and the long-chain fatty acids 20∶1 and 20∶2, which increased to more than 10-fold of their initial level in accord with oil deposition. The above processes were associated with the first rise in VOCs production (Figure S3), in accordance with the recently revealed VOC patterns of developmental change for the same material as that used in this study [7].

**Table 1:** Fixed oil composition of *Nigella sativa* at 50 DAA and 82 DAA seeds.

Fatty acid	% of TFA at 50 DAA	% of TFA at 82 DAA
14∶0	0.2±0.01	0.3±0.03
16∶0	12.5±0.19	13.2±0.51
16∶1	0.3±0.01	0.3±0.01
16∶3	0.1±0.01	0.14±0.01
18∶0	2.6±0.10	2.6±0.07
18∶1 n–9	21.9±0.39	20.9±1.13
18∶1 n–7	0.5±0.19	0.7±0.19
18∶2	58.4±0.76	58.5±0.30
18∶3 n–3	0.5±0.04	0.3±0.03
20∶0	0.1±0.01	0.2±0.01
20∶1	0.3±0.02	0.3±0.01
20∶2	2.3±0.06	2.6±0.18

Means of three independent determinations originating from three separate plants each±SE. Fatty acid composition is presented as relative percentage (%) of total fatty acid and was analyzed by GC-FID after their transesterification with 2% H_2_SO_4_ in dry methanol. Identification was accomplished by comparison of sample peak retention times with those of FAME standard mixtures.

The content of TFA increased between 35–50 DAA, reaching an apex at 50 DAA indicating the accumulation of storage triacylglycerols (TAG). Later in the time course of seed maturation (50 to 80 DAA), TFA content decreased significantly, and the final content amounted to 60 to 70% of the maximal value, with the composition being retained (Table 1). The here-described trend was observed for the absolute contents of the major fatty acids (16∶0, 18∶0, 18∶2, 18∶1n–9). A different pattern of change was shown for C_16_ unsaturated fatty acids and for 18∶3n-3, which were present in low levels in mature seeds. A coordinated decrease in TAG-associated fatty acids [36] occurred after 50 DAA, particularly in linoleic acid (18∶2), with this decrease being associated with the accumulation of VOCs (Figure S3).

### Directed Network Analysis Highlights Coordinated Metabolic Shifts

To understand the relationships between the metabolic processes occurring during the development of *Nigella* seeds, time-resolved profiles of volatiles, of central metabolism compounds, and of fatty acids were subjected to network-based analysis. To create directed network edges, we used the asymmetric similarity measure iota [24] (see Materials and Methods) in combination with a threshold value ensuring statistical soundness; here, the threshold was selected to guarantee a false discovery rate of 5%, corresponding to a threshold value of 0.995. A directed edge is indicative of the dependence of the originating on the receiving node, which may indicate either a regulation-associated association or a product-substrate relationship. In both cases, monotonous changes in the time series profile attributed to the receiver node are expected to relate to a monotonous change in the profile of the originator node. Following this approach, the resulting network contains 107 nodes, corresponding to the annotated metabolites, connected by 1087 directed edges. The relative densities of this directed network (compared to all possible edges that could be established on the given number of nodes) was 0.0958. The diameter of the network, i.e., the length of the longest of all the shortest paths connecting any two nodes, was equal to 7, suggesting the presence of denser subnetworks.

**Figure 5:**
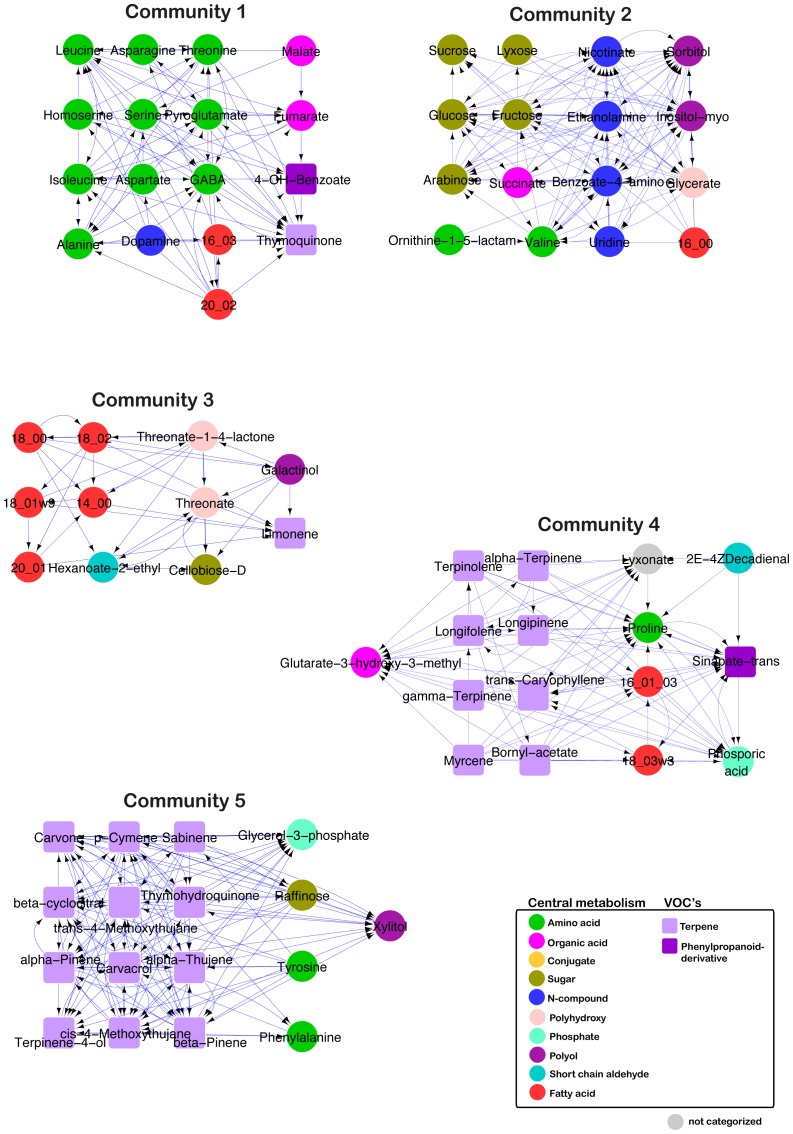
*Nigella* communities. Shown are the *Nigella* communities, where nodes display higher connectivity to each other than to the rest of the network. The communities were generated using the walktrap-community algorithm. Their stability was confirmed by robustness analysis. Only communities with more than one node are illustrated.

To characterize the locally dense parts of the obtained network suggesting coordinated changes in metabolic content, we next identified network communities. A network community corresponds to a sub-network of nodes that are more connected between each other than with other nodes in the network (Figure 5). We used the walk-trap community algorithm, and found five communities by bounding their size between 2 to 20 nodes. Robustness analysis was conducted to test the stability of the identified communities (see Materials and Methods).

Generally, the network communities highlighted both the tight coordination between metabolite classes and the crosstalk between central and specialized metabolisms. Figure 5 shows the communities containing more than one node. Community 1 is enriched with amino acids and organic acids, most of which show crosstalk (i.e., in-coming and out-going edges) with each other. Fumarate and GABA have an increased number of in-coming edges, while 4-OH-benzoate displays a high number of out-going edges. Thymoquinone, the only volatile in this community, has mostly in-coming edges.

Community 2 is predominantly characterized by sugars; sucrose has mainly out-going edges, while the other sugars have mainly incoming edges, suggesting that sucrose is the source and probable regulator of the biosynthesis of other sugars. The rest of the community is composed of a mixture of different compound classes of the central metabolism, i.e., amino acids, organic acids, polyols, N-compounds, polyhydroxy compounds and a 16∶00 fatty acid. This fatty acid has only in-coming edges. Community 2 does not contain any volatiles.

Community 3 is enriched with fatty acids. Community 4 represents the transitory stage between central metabolism and VOCs, reflected by a balanced distribution of central metabolites and volatiles. In this community, the central metabolites, proline, lyxonate, phosphoric acid, sinapate and glutarate-3-hydroxy, are clearly receiving nodes. Community 5 is characterized by the predominant presence of terpenes and terpenoids, all showing a balanced crosstalk with each other. Similar to Community 4, in Community 5 xylitol and glycerol–3-phosphate are sink nodes, having only in-coming edges, a finding that highlights the interplay between the components of central metabolism and the VOCs. Raffinose appears to occupy a transitory position in this Community, with a balanced number of in- and out-going edges.

## Discussion

To date, studies of the metabolic processes occurring during seed development and maturation have largely been dedicated to the understanding of the accumulation of storage reserves of proteins, starch or TAGs [6], to the imposition of dormancy, and to the acquisition of desiccation tolerance, processes to which the maturation of orthodox seeds is indeed dedicated [37]. Nonetheless, during desiccation seeds can recycle a significant percentage of storage reserves [38] and accumulate unbound metabolites [17] to sustain long-term storage of reserves and in preparation for the early events of germination [2]. Against this background, the phenomenon of orthodox true seeds accumulating volatiles during maturation has not received adequate attention. By using developing *Nigella* seeds as a model system, we investigated the central metabolic processes in VOC-producing seeds and the interaction between VOCs and central metabolites during seed development.

### *Nigella* Seed Development is Characterized by Two Key Metabolic Shifts

The integration of the data from the current analysis of central metabolites and acyl moieties of complex lipids [determined as fatty acid methyl esters (FAMEs)] with volatile profiles measured in our earlier study [7] revealed that *Nigella* metabolism undergoes two metabolic shifts during the transition of the seed from development to photosynthetic maturation and pigmented desiccation. PCA analysis and model-based clustering suggest that, from a metabolic standpoint, *Nigella* development can be divided into three phases during which the seeds exhibit considerable differences in metabolism, as controlled by two shifts in C–N metabolism. The first shift during the maturation of the seed was marked by significant changes in the levels of metabolites such as sugars and sugar alcohols and precursors of TAG metabolism; these findings suggest intensive activity in storage resource accumulation and glycolysis to support the production of fatty acids (Figure 4) and their incorporation in to TAGs. Outstanding among the amino acids, Asn and the N-containing compound dopamine were found to significantly, but transiently, increase during this period, reflecting their role as key sources of N nutrition for the developing seeds. For Asn, these findings are in keeping with the peak intake from the phloem to the soluble nitrogen pool in white lupin developing seeds [39]. The second shift was characterized by a reduction in hexoses, sugar alcohols and fatty acids. Shikimate and precursors of the secondary shikimate metabolism 3,4-dihydroxyphenyl-acetate and dopamine, were also decreased at this stage, probably reflecting their incorporation into downstream secondary metabolic pathways. Initiation of VOC biosynthesis at this stage partly supports this suggestion. Raffinose accumulation follows the earlier accumulation of galactinol, the galactosyl donor for the biosynthesis of raffinose family oligosaccharides (RFO) [40]. Raffinose and galactinol – along with sucrose – are desiccation-related compounds, which have long been known to be involved (by contributing to the formation of a glassy matrix) in the structural acquisition of desiccation tolerance [41]–[43] in orthodox seeds of different plant species [17], [44]. These sugars provide also a carbon pool at the beginning of germination [16].

### Amino Acid Pattern of Change Suggests Intermittent N Metabolism Rearrangement. Could it Sustain N-containing Secondary Compounds Biosynthesis?

In *Nigella*, amino acids initially accumulated during early development of the seed, possibly reflecting input of N-containing compounds from the phloem; thereafter they decreased, either gradually or abruptly, probably due to their incorporation into storage proteins. Our results show fluctuations of Phe and Trp concentrations during seed development, with the values increasing slightly above their median contents at 20–25 DAA, 43–46 DAA and during desiccation. Such “waves” of accumulation are common for most amino acids, albeit to different extents. In this study, we found the accumulation of amino acids between 25 and 30 DAA to be rather general (although a marked 10-to 100-fold change was observed in the contents of Tyr, Ser, Ile and Leu, Ala and beta-Ala) and was probably associated with the earlier increase in Gln and Asn, and/or the recycling of N from ornithine and Pro, the content of the latter decreasing 100 times between 0 and 20 DAA. During later waves of increases in the concentrations of N-compounds, a sequential accumulation of Phe/Trp and an associated increase of the chorismate-derivative 3,4-dihydroxyphenyl-acetate were observed. In parallel to increased shikimate metabolism, dopamine increased transiently between 35 and 55 DAA. Dopamine is the product of decarboxylation of L-DOPA (3,4-dihydroxy-phenylalanine), which is governed by Tyr decarboxylase (TYDC) downstream of the shikimate pathway. Noteworthy, directed network analysis generated communities including shikimate amino acids Phe, Tyr together with terpenes and terpenoids. The analysis also linked between Leu derived kaempferol-3-o-glucopyranoside-6-(also known as 3-hydroxy-3-methylglutarate) and terpenes. These lines of evidence strongly call for more work on the contribution of amino acids to *Nigella* seed volatiles.

### C Partitioning Toward VOC Biosynthesis

Profiling and quantification of fatty acids in *Nigella* seeds showed that TFA accumulation occurred during early-mid maturation, probably due to biosynthesis and deposition of storage TAGs in the developing seeds. Later in development, the levels of TFA decreased by about 30% of their maximum accumulation (Figure 4). Malate, a major precursor of fatty acid biosynthesis in the heterotrophic plastid of the developing seed [45], increased during early maturation (25 DAA), preceding the rise in TFA content, and decreased abruptly at mid maturation (35 DAA) and again at the onset of desiccation (60 DAA).

These significant changes in pattern of fatty acids during maturation could be representative of C repartitioning toward the biosynthesis of secondary metabolites. Indeed, polyunsaturated fatty acid catabolism can lead to the direct production of a series of volatiles, including the volatile aldehydes. However, an indirect relation between TAG and volatiles is more likely in *Nigella* seeds. For example, terpenoids, such as carvacrol, are expensive (in carbon “currency”) to produce due to their chemical reduction and the need for dedicated enzymes [46]. Moreover, the terpenoids are synthesized from acetyl CoA units hence providing a competitive metabolic process to TAG production. Among the fatty acids measured, we observed a continuous stepwise reduction of linolenic acid 18∶3n-3, a substrate of LOX, which in the network analysis clustered tightly within the volatile module (community 4). LOXes catalyze the regio- and stereo-specific dioxygenation of PUFAs (18∶2 and 18∶3) and are involved in many different developmental processes, including the production of plant specific volatiles [47]. LOX has been shown to initiate the mobilization of TAGs in germinating cucumber seeds and to initiate the production of volatile aldehydes [48]. This supposedly dual role of fatty acids during the development of *Nigella* seeds is reflected in: (i) the developmentally alternated accumulation of fatty acid and VOCs, and (ii) the association between fatty acids and VOCs in the community analysis of the directed network.

### Directed Network Analysis Identifies Coordinated Processes during *Nigella* Seed Development and Possible Metabolic Dependencies

When analyzing the data via directed network analysis, we identified significant relationships between metabolites closely related to precursors of known biochemical pathway for VOC biosynthesis. In the communities enriched with VOCs, the metabolites xylitol and glycerol-3-phosphate were characterized by incoming edges. These metabolites are closely related forms of precursors of the chloroplastic deoxyxylulose phosphate pathway, i.e. the mevalonate-independent pathway in the plastid, for VOC biosynthesis [12]. The analysis also confirmed the coordinated pattern of change between amino acids, sugars, fatty acids and volatiles, each enriching a different community. That having been said, the directionality of the edges, being based on a mathematical measure, is not trivially interpretable. In-coming edges suggest a regulatory dependence of the originator node on the receiver node or, on a temporal scale, a product and precursor relationship, respectively. However, such relationships cannot be obtained by the classically used measures, such as correlations, which usually fall in the category of symmetric measures. For example, the incoming edges on sucrose suggest the dependence of the other sugars on the sucrose pool, similar to the example given above for the chloroplastic deoxyxylulose phosphate pathway for VOC biosynthesis. However, more difficult to interpret are the numerous incoming links from amino acids to thymoquinone. Importantly, within communities shared between central metabolites and VOCs, the relation usually involved central metabolites acting as receiving nodes, suggesting a dependence of VOCs on central intermediates. Nevertheless, beyond this generalized statement, a more specific interpretation of these results is not possible and would require further methodology-related developments.

**Figure 6:**
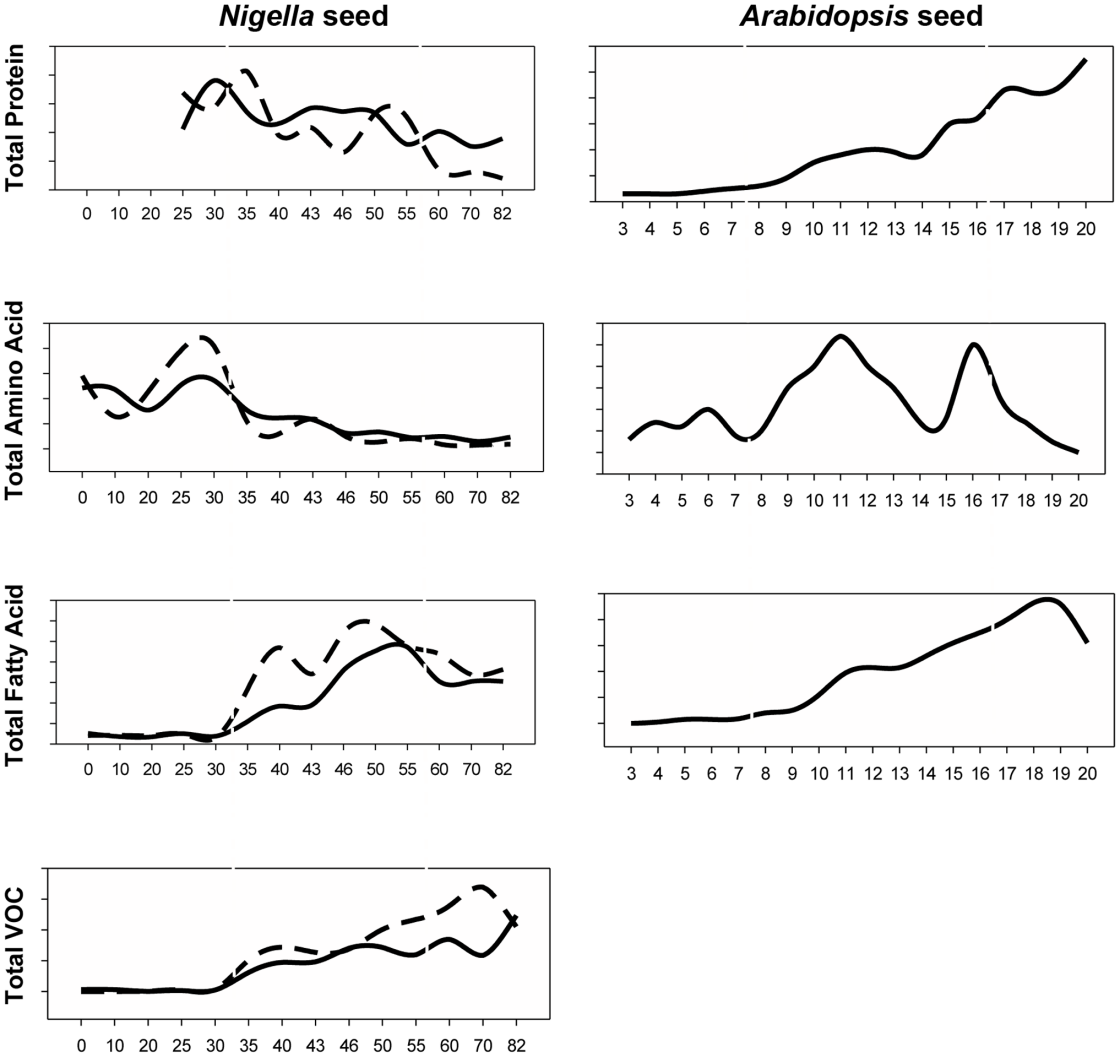
Comparative analysis of dynamic regulation of main compositions during seed development in *Arabidopsis* and *Nigella*. Total amino acid is the sum of detected free amino acids. The unit for total protein content is µg/seed, all other compositions use ng/seed as unit. Data came from reported paper [16].

Finally, Figure 6 presents a schematic comparison of the broad developmental patterns of metabolite changes in abundance between current knowledge of *Arabidopsis* seeds and the current data for *Nigella* seeds. A striking difference is evident in the patterns of change and the major reductions in sucrose, fatty acids and proteins during *Nigella* seed maturation. While to some extent speculative, it is tempting to suggest a link between these differences and the metabolic investment in VOC production. Future work could test this hypothesis by using metabolic flux analysis during *Nigella* seed development.

In conclusion, the results of metabolite profiling and directed network analysis presented here suggest that in *Nigella*, major degradation of fatty acids and N-compounds provides the building blocks for the biosynthesis of volatiles, as is known to be the case in several other plant species [11]. Amino acids, especially the aromatic amino acids, branched chain amino acids and methionine serve as precursors for many aroma volatiles in fruits [49], [50]. While these volatiles are generally absent in *Nigella*, an indirect metabolic link probably exists, e.g. via Leu derived kaemperol glycoside (glutarate 3 hydroxy 3 methyl in community 4). Network analysis inferred a link between fatty acids and fatty-acid-derived volatile caproic acid (2-ethylhexanoate) [51]. Moreover, the analysis supported the involvement of the LOX substrate fatty acid 18∶3n-3 and metabolites of the central metabolism closely related to terpenoid precursors [52] in the biosynthesis of volatiles during *Nigella* seed maturation. Future work should explore the biological meaning of directionality in iota-based networks, however it is safe to suggest that the direction of the edge is at least to some extent a representation of metabolic dependence, e.g. carbon metabolism is largely dependent upon sucrose pools as suggested by the edges directed toward the latter metabolite in community 2. Finally, seed VOCs have been associated with the regulation of germination [53], with plant/pathogen interactions [54]–[56], and with structure of pest communities [57]. Yet, our understanding of the role of VOC role in seeds is limited. The present study shows that VOC-producing seeds probably repartition their C–N metabolism during the stage of VOC production. Further, functional research on VOC-producing seeds is required to address the open questions still remaining.

## Supporting Information

Figure S1 **Number of clusters for k-means clustering with highest probability (in red) for** ***Nigella EH*** **.**
(DOC)

Figure S2 **The concept of the iota measure.**
(PDF)

Figure S3 **Relative content of volatiles of** ***Nigella*** **seeds (see legend).** The heatmap is an elaboration of data published in Botnick et al., 2012, and it is here presented with permission of the authors.
(DOC)

Table S1 **Relative metabolite content from GC-MS analysis of** ***Nigella sativa*** **EH accession.** The data is here presented as normalized metabolite content on internal standard ribitol. Three biological replica, each consisting of 20 mg seeds from 3 individual plants, were used at each time point from anthesis (0 DAA) to seed maturity (82 DAA).
(XLS)

Table S2 **Eigenvectors values (in descending order) of metabolites were calculated by PCA algorithm for 1st, 2nd and 3rd components of EH genotype.**
(DOC)

Table S3 **Metabolites that level changed significantly during the metabolic shifts of** ***Nigella*** **seed development process.** Trend of change is presented in brackets. Results were detected by SSSTP with confidence interval 95% and FDR correction
(CSV)

## References

[1] Holdsworth MJ, Bentsink L, Soppe WJJ (2008) Molecular networks regulating Arabidopsis seed maturation, after-ripening, dormancy and germination. New Phytologist 179: 33–54. 18422904 10.1111/j.1469-8137.2008.02437.x

[2] Angelovici R, Galili G, Fernie AR, Fait A (2010) Seed desiccation: a bridge between maturation and germination. Trends in Plant Science 15: 211–218. 20138563 10.1016/j.tplants.2010.01.003

[3] Weber H, Borisjuk L, Wobus U (2005) Molecular Physiology of Legume Seed Development. Annual Review of Plant Biology 56: 253–279. 10.1146/annurev.arplant.56.032604.144201 15862096

[4] Bennett RN, Wallsgrove RM (1994) Secondary metabolites in plant defence mechanisms. New Phytologist 127: 617–633. 10.1111/j.1469-8137.1994.tb02968.x 33874382

[5] Fahn A (1982) Plant anatomy. Oxford: Pergamon Press.

[6] Wobus U, Weber H (1999) Sugars as Signal Molecules in Plant Seed Development. Biological Chemistry 380: 937–944. 10494845 10.1515/BC.1999.116

[7] Botnick I, Xue W, Bar E, Ibdah M, Schwartz A, et al (2012) Distribution of Primary and Specialized Metabolites in Nigella sativa Seeds, a Spice with Vast Traditional and Historical Uses. Molecules 17: 10159–10177. 22922285 10.3390/molecules170910159 PMC6268483

[8] Houghton PJ, Zarka R, de las Heras B, Hoult JRS (1995) Fixed Oil of Nigella sativa and Derived Thymoquinone Inhibit Eicosanoid Generation in Leukocytes and Membrane Lipid Peroxidation. Planta Med 61: 33–36. 7700988 10.1055/s-2006-957994

[9] Benkaci–Ali F, Baaliouamer A, Meklati BY, Chemat F (2007) Chemical composition of seed essential oils from Algerian Nigella sativa extracted by microwave and hydrodistillation. Flavour and Fragrance Journal 22: 148–153.

[10] Burits M, Bucar F (2000) Antioxidant activity of Nigella sativa essential oil. Phytotherapy Research 14: 323–328. 10925395 10.1002/1099-1573(200008)14:5<323::aid-ptr621>3.0.co;2-q

[11] Schwab W, Davidovich-Rikanati R, Lewinsohn E (2008) Biosynthesis of plant-derived flavor compounds. The Plant Journal 54: 712–732. 18476874 10.1111/j.1365-313X.2008.03446.x

[12] Davis E, Croteau R (2000) Cyclization Enzymes in the Biosynthesis of Monoterpenes, Sesquiterpenes, and Diterpenes. In: Leeper F, Vederas J, editors. Biosynthesis: Springer Berlin Heidelberg. 53–95.

[13] Marcel D, Joop JAvL, Roxina S (2009) Chemical complexity of volatiles from plants induced by multiple attack. Nature Chemical Biology 5: 317–324. 19377458 10.1038/nchembio.169

[14] Zhang J, Wang X, Yu O, Tang J, Gu X, et al (2011) Metabolic profiling of strawberry (Fragariaananassa Duch.) during fruit development and maturation. Journal of Experimental Botany 62: 1103–1118. 21041374 10.1093/jxb/erq343

[15] Borisjuk L, Rolletschek H, Wobus U, Weber H (2003) Differentiation of legume cotyledons as related to metabolic gradients and assimilate transport into seeds. Journal of Experimental Botany 54: 503–512. 12508061 10.1093/jxb/erg051

[16] Baud S, Boutin JP, Miquel M, Lepiniec L, Rochat C (2002) An integrated overview of seed development in Arabidopsis thaliana ecotype WS. Plant Physiology and Biochemistry 40: 151–160.

[17] Fait A, Angelovici R, Less H, Ohad I, Urbanczyk-Wochniak E, et al (2006) Arabidopsis Seed Development and Germination Is Associated with Temporally Distinct Metabolic Switches. Plant Physiology 142: 839–854. 16963520 10.1104/pp.106.086694 PMC1630763

[18] Mounet F, Lemaire-Chamley M, Maucourt M, Cabasson C, Giraudel J-L, et al (2007) Quantitative metabolic profiles of tomato flesh and seeds during fruit development: complementary analysis with ANN and PCA. Metabolomics 3: 273–288.

[19] Thompson R, Burstin J, Gallardo K (2009) Post-Genomics Studies of Developmental Processes in Legume Seeds. Plant Physiology 151: 1023–1029. 19675147 10.1104/pp.109.143966 PMC2773076

[20] Steuer R, Kurths J, Fiehn O, Weckwerth W (2003) Observing and interpreting correlations in metabolomic networks. Bioinformatics 19: 1019–1026. 12761066 10.1093/bioinformatics/btg120

[21] Urbanczyk-Wochniak E, Willmitzer L, Fernie AR (2006) Integrating Profiling Data. 77–85. 10.1007/978-1-59745-244-1_5 17035681

[22] Toubiana D, Fernie AR, Nikoloski Z, Fait A (2013) Network analysis: tackling complex data to study plant metabolism. Trends in Biotechnology 31: 29–36. 23245943 10.1016/j.tibtech.2012.10.011

[23] Schreiber T (2000) Measuring Information Transfer. Physical Review Letters 85: 461–464. 10991308 10.1103/PhysRevLett.85.461

[24] Hempel S, Koseska A, Nikoloski Z, Kurths J (2011) Unraveling gene regulatory networks from time-resolved gene expression data – a measures comparison study. BMC Bioinformatics 12: 292. 21771321 10.1186/1471-2105-12-292 PMC3161045

[25] Bradford MM (1976) A rapiad and sensitive method for the quantitation of microgram quantities of protein utilizing the principle of protein-dye binding. Anal Biochem 72: 248–254. 942051 10.1016/0003-2697(76)90527-3

[26] McKinney (1941) Absorption of light by chlorophyll solutions. J Biol Chem 140: 315–332.

[27] Lisec J, Schauer N, Kopka J, Willmitzer L, Fernie AR (2006) Gas chromatography mass spectrometry-based metabolite profiling in plants. Nat Protocols 1: 387–396. 17406261 10.1038/nprot.2006.59

[28] Roessner U, Luedemann A, Brust D, Fiehn O, Linke T, et al (2001) Metabolic Profiling Allows Comprehensive Phenotyping of Genetically or Environmentally Modified Plant Systems. The Plant Cell Online 13: 11–29. 10.1105/tpc.13.1.11 PMC2652711 11158526

[29] Erban A, Schauer N, Fernie AR, Kopka J (2006) Nonsupervised Construction and Application of Mass Spectral and Retention Time Index Libraries From Time-of-Flight Gas Chromatography-Mass Spectrometry Metabolite Profiles. 19–38. 10.1007/978-1-59745-244-1_2 17035678

[30] Hummel J, Selbig J, Walther D, Kopka J (2007) The Golm Metabolome Database: a database for GC-MS based metabolite profiling. In: Nielsen J, Jewett M, editors. Metabolomics: Springer Berlin Heidelberg. 75–95.

[31] Cohen Z, Reungjitchachawali M, Siangdung W, Tanticharoen M (1993) Production and partial purification of γ-linolenic acid and some pigments fromSpirulina platensis. Journal of Applied Phycology 5: 109–115.

[32] Saeed A, Sharov V, White J, Li J, Liang W, et al (2003) TM4: a free, open-source system for microarray data management and analysis. Biotechniques 34: 374–378. 12613259 10.2144/03342mt01

[33] Sokal RR, Rohlf FJ (1994) Biometry. New York: W. H. Freeman and Company. 887 p.

[34] Hempel S, Koseska A, Nikoloski Z (2013) Data-driven reconstruction of directed networks. The European Physical Journal B 86: 1–17.

[35] Rosnoblet C, Aubry C, Leprince O, Vu BL, Rogniaux H, et al (2007) The regulatory gamma subunit SNF4b of the sucrose non-fermenting-related kinase complex is involved in longevity and stachyose accumulation during maturation of Medicago truncatula seeds. The Plant Journal 51: 47–59. 17488238 10.1111/j.1365-313X.2007.03116.x

[36] Spiteller P, Kern W, Reiner J, Spiteller G (2001) Aldehydic lipid peroxidation products derived from linoleic acid. Biochimica et Biophysica Acta (BBA) - Molecular and Cell Biology of Lipids 1531: 188–208. 11325611 10.1016/s1388-1981(01)00100-7

[37] Baud S, Lepiniec L (2010) Physiological and developmental regulation of seed oil production. Progress in Lipid Research 49: 235–249. 20102727 10.1016/j.plipres.2010.01.001

[38] Chia TYP, Pike MJ, Rawsthorne S (2005) Storage oil breakdown during embryo development of Brassica napus (L.). Journal of Experimental Botany 56: 1285–1296. 15767324 10.1093/jxb/eri129

[39] Desmaison AM, Marcher MH, Tixier M (1984) Changes in the free and total amino acid composition of ripening chestnut seeds. Phytochemistry 23: 2453–2456.

[40] Sprenger N, Keller F (2000) Allocation of raffinose family oligosaccharides to transport and storage pools in Ajuga reptans: the roles of two distinct galactinol synthases. The Plant Journal 21: 249–258. 10758476 10.1046/j.1365-313x.2000.00671.x

[41] Castillo EM, De Lumen BO, Reyes PS, De Lumen HZ (1990) Raffinose synthase and galactinol synthase in developing seeds and leaves of legumes. Journal of Agricultural and Food Chemistry 38: 351–355.

[42] Saravitz DM, Pharr DM, Carter TE (1987) Galactinol Synthase Activity and Soluble Sugars in Developing Seeds of Four Soybean Genotypes. Plant Physiology 83: 185–189. 16665199 10.1104/pp.83.1.185 PMC1056321

[43] Brenac P, Horbowicz M, Downer SM, Dickerman AM, Smith ME, et al (1997) Raffinose accumulation related to desiccation tolerance during maize (Zea mays L.) seed development and maturation. Journal of Plant Physiology 150: 481–488.

[44] Buitink J, Leprince O (2008) Intracellular glasses and seed survival in the dry state. Comptes Rendus Biologies 331: 788–795. 18926493 10.1016/j.crvi.2008.08.002

[45] Pleite R, Pike MJ, Garcés R, Martínez-Force E, Rawsthorne S (2005) The sources of carbon and reducing power for fatty acid synthesis in the heterotrophic plastids of developing sunflower (Helianthus annuus L.) embryos. Journal of Experimental Botany 56: 1297–1303. 15767323 10.1093/jxb/eri130

[46] Gershenzon J (1994) Metabolic costs of terpenoid accumulation in higher plants. Journal of Chemical Ecology 20: 1281–1328. 24242341 10.1007/BF02059810

[47] Liavonchanka A, Feussner I (2006) Lipoxygenases: Occurrence, functions and catalysis. Journal of Plant Physiology 163: 348–357. 16386332 10.1016/j.jplph.2005.11.006

[48] Weichert H, Kolbe A, Kraus A, Wasternack C, Feussner I (2002) Metabolic profiling of oxylipins in germinating cucumber seedlings – lipoxygenase-dependent degradation of triacylglycerols and biosynthesis of volatile aldehydes. Planta 215: 612–619. 12172844 10.1007/s00425-002-0779-4

[49] Gonda I, Bar E, Portnoy V, Lev S, Burger J, et al (2010) Branched-chain and aromatic amino acid catabolism into aroma volatiles in Cucumis melo L. fruit. Journal of Experimental Botany 61: 1111–1123. 20065117 10.1093/jxb/erp390 PMC2826658

[50] Gonda I, Lev S, Bar E, Sikron N, Portnoy V, et al (2013) Catabolism of l–methionine in the formation of sulfur and other volatiles in melon (Cucumis melo L.) fruit. The Plant Journal 74: 458–472. 23402686 10.1111/tpj.12149

[51] Jardine K, Barron-Gafford GA, Norman JP, Abrell L, Monson RK, et al (2012) Green leaf volatiles and oxygenated metabolite emission bursts from mesquite branches following light-dark transitions. Photosynthesis Research 113: 321–333. 22711426 10.1007/s11120-012-9746-5

[52] Lichtenthaler HK (1999) THE 1-DEOXY-D-XYLULOSE-5-PHOSPHATE PATHWAY OF ISOPRENOID BIOSYNTHESIS IN PLANTS. Annual Review of Plant Physiology and Plant Molecular Biology 50: 47–65. 10.1146/annurev.arplant.50.1.47 15012203

[53] Holm RE (1972) Volatile Metabolites Controlling Germination in Buried Weed Seeds. Plant Physiology 50: 293–297. 16658159 10.1104/pp.50.2.293 PMC366127

[54] Unsicker SB, Kunert G, Gershenzon J (2009) Protective perfumes: the role of vegetative volatiles in plant defense against herbivores. Current Opinion in Plant Biology 12: 479–485. 19467919 10.1016/j.pbi.2009.04.001

[55] Moyes CL, Raybould AF (2001) The role of spatial scale and intraspecific variation in secondary chemistry in host–plant location by Ceutorhynchus assimilis (Coleoptera: Curculionidae). Proceedings of the Royal Society of London Series B: Biological Sciences 268: 1567–1573. 11487403 10.1098/rspb.2001.1685 PMC1088779

[56] Bruce TJA, Wadhams LJ, Woodcock CM (2005) Insect host location: a volatile situation. Trends in plant science 10: 269–274. 15949760 10.1016/j.tplants.2005.04.003

[57] Newton E, Bullock J, Hodgson D Glucosinolate polymorphism in wild cabbage (Brassica oleracea) influences the structure of herbivore communities. Oecologia 160: 63–76. 19214588 10.1007/s00442-009-1281-5

