# Supplementary material for: The Investment in Scent: Time-Resolved Metabolic Processes in Developing Volatile-Producing Nigella sativa L. Seeds
Source: PLoS One. 2013 Sep 3;8(9):e73061. doi: 10.1371/journal.pone.0073061 (PMC3760832; doi:10.1371/journal.pone.0073061)
Supplement: Figure S1 — Number of clusters for k-means clustering with highest probability (in red) for Nigella EH . (DOC) [file pone.0073061.s001.doc]

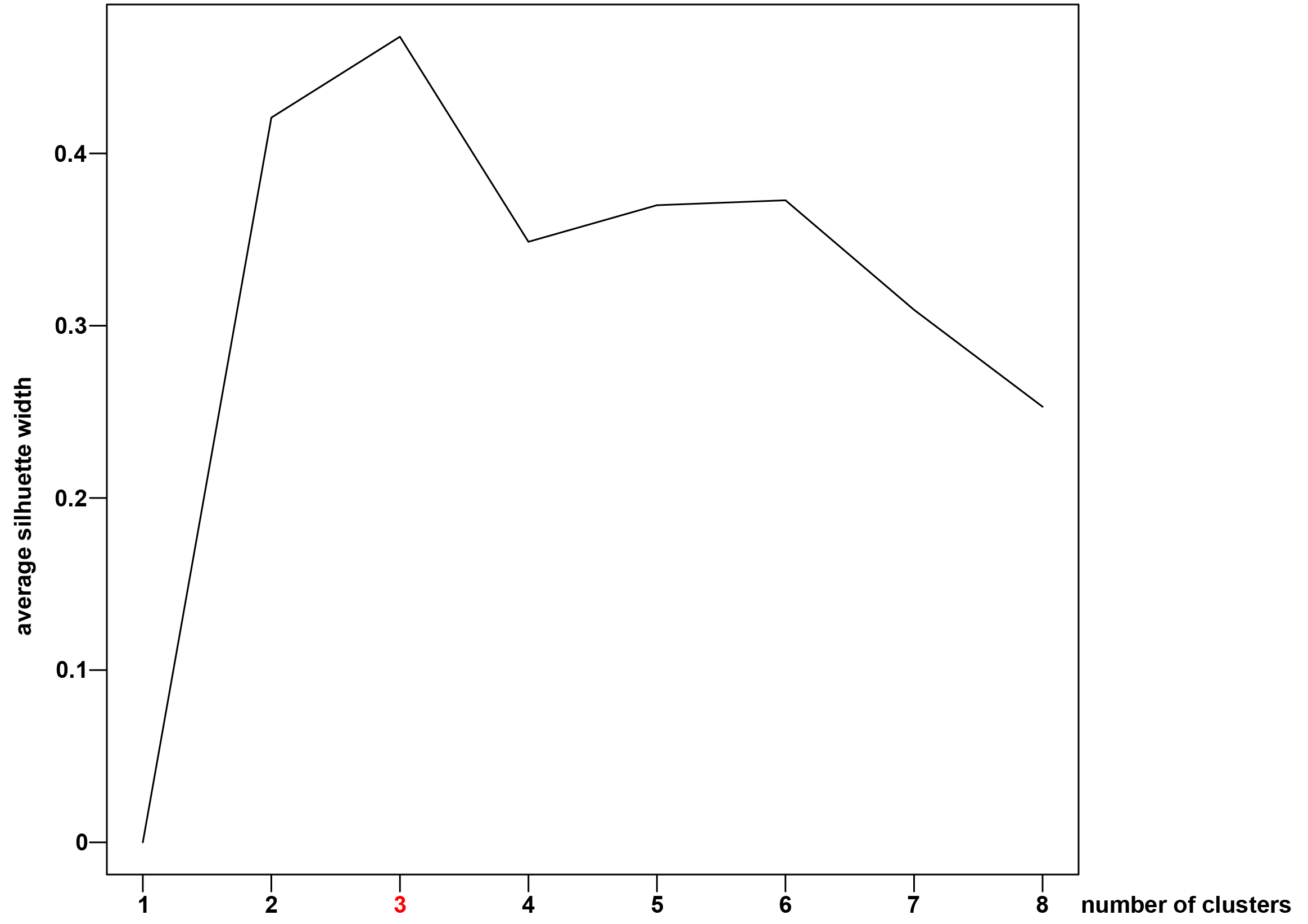


**Figure S1.** Number of clusters for k-means clustering with highest probability (in red) for *Nigella EH*
