# Supplementary material for: The Investment in Scent: Time-Resolved Metabolic Processes in Developing Volatile-Producing Nigella sativa L. Seeds
Source: PLoS One. 2013 Sep 3;8(9):e73061. doi: 10.1371/journal.pone.0073061 (PMC3760832; doi:10.1371/journal.pone.0073061)
Supplement: Figure S2 — The concept of the iota measure. (PDF) [file pone.0073061.s002.pdf]

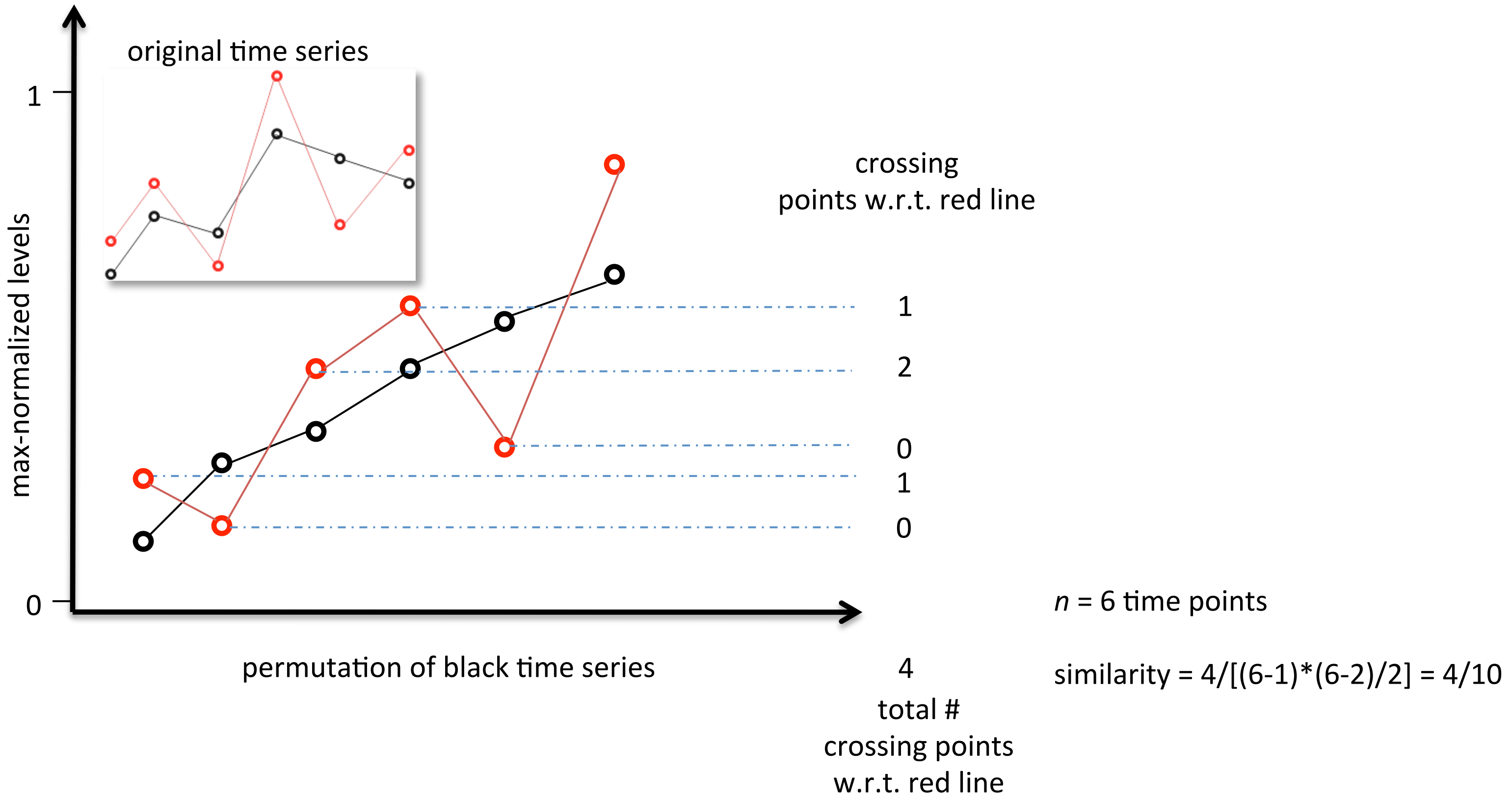

Figure S2. The concept of the iota measure. Given are two time series depicted in the upper left corner (inlay). We assume that the time series are max-normalized (by dividing their entries by the respective maximum values), as shown on the y-axis. The x-axis corresponds to the permutation which transforms the black time series into monotonically non-decreasing (compare to the inlay). The same permutation is used to reorder the red time series. The effect on the montonocity of the red series is quantified by counting the number of crossings (with respect to the red line itself) obtained by drawing lines to the right of each point. For instance, point 1 has a single crossing, while point 3 has two crossing. The final score is the total number of crossings normalized by the maximum number of crossings in a time series of n = 6 time points (which amounts to (n-1)(n-2) = 10).
