# Supplementary material for: The Investment in Scent: Time-Resolved Metabolic Processes in Developing Volatile-Producing Nigella sativa L. Seeds
Source: PLoS One. 2013 Sep 3;8(9):e73061. doi: 10.1371/journal.pone.0073061 (PMC3760832; doi:10.1371/journal.pone.0073061)
Supplement: Figure S3 — Relative content of volatiles of Nigella seeds (see legend). The heatmap is an elaboration of data published in Botnick et al., 2012, and it is here presented with permission of the authors. (DOC) [file pone.0073061.s003.doc]

**
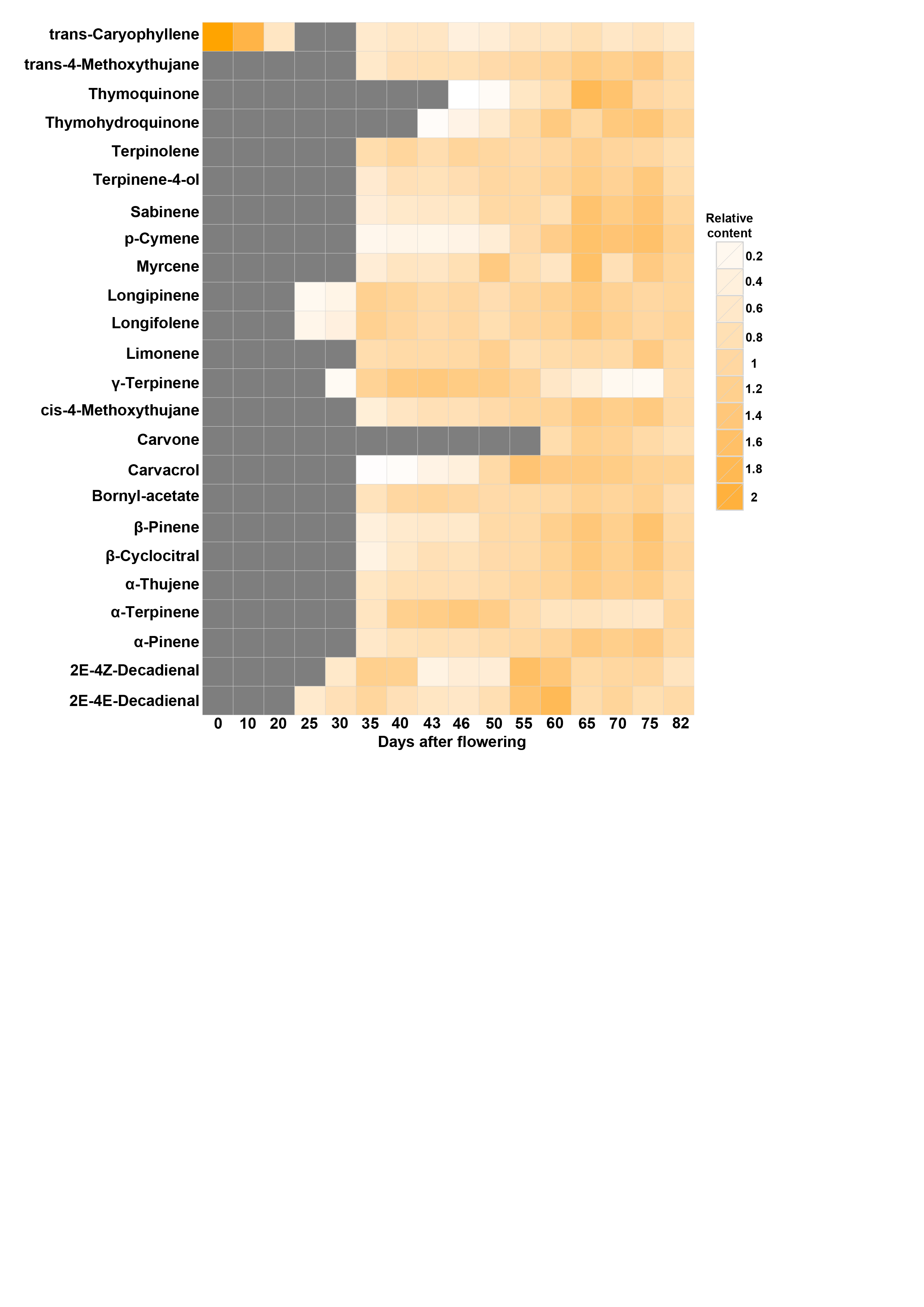
**

**Figure S3.** Relative content of volatiles of *Nigella* seeds (see legend). The heatmap is an elaboration of data published in Botnick et al., 2012, and it is here presented with permission of the authors
