# Supplementary material for: The Investment in Scent: Time-Resolved Metabolic Processes in Developing Volatile-Producing Nigella sativa L. Seeds
Source: PLoS One. 2013 Sep 3;8(9):e73061. doi: 10.1371/journal.pone.0073061 (PMC3760832; doi:10.1371/journal.pone.0073061)
Supplement: Table S2 — Eigenvectors values (in descending order) of metabolites were calculated by PCA algorithm for 1st, 2nd and 3rd components of EH genotype. (DOC) [file pone.0073061.s005.doc]

| **Metabolite** | **1^st^ PC** | **Metabolite** | **2^nd^ PC** | **Metabolite** | **3^rd^ PC** |
| --- | --- | --- | --- | --- | --- |
| Glycerate | 2.9 | Dopamine | 4.1 | Dopamine | 3.2 |
| Galactinol | 2.3 | Gln | 3.0 | Pro | 2.7 |
| Raffinose | 2.1 | Acetate-3,4-dihydroxyphenyl | 2.9 | Tyr | 2.5 |
| Acetate-3,4-dihydroxyphenyl | 2.1 | Glycerate | 2.6 | Galactinol | 2.3 |
| Succinate | 2.0 | Malate | 2.3 | Ser | 1.7 |
| Ser | 2.0 | GABA | 2.3 | Raffinose | 1.7 |
| Dopamine | 1.9 | Fumarate | 2.0 | Trp | 1.5 |
| Nicotinate | 1.8 | Fructose | 2.0 | GABA | 1.4 |
| Cellobiose | 1.7 | beta_Ala | 1.9 | Glycerophosphoglycerol | 1.3 |
| Pro | 1.5 | Shikimate | 1.7 | Fumarate | 1.3 |

**Table S4.** Eigenvectors values (in descending order) of metabolites were calculated by PCA algorithm for 1^st^, 2^nd^ and 3^rd^ components of *EH* genotype
